# Heavy metal music meets complexity and sustainability science

**DOI:** 10.1186/s40064-016-3288-9

**Published:** 2016-09-21

**Authors:** David G. Angeler

**Affiliations:** Department of Aquatic Sciences and Assessment, Swedish University of Agricultural Sciences, Box 7050, 750 07 Uppsala, Sweden

**Keywords:** Auditory arts, Complexity science, Complex adaptive systems, Heavy metal music, Social-ecological systems, Sustainability, Transdisciplinary science, Environmental education, Global change

## Abstract

This paper builds a bridge between heavy metal music, complexity theory and sustainability science to show the potential of the (auditory) arts to inform different aspects of complex systems of people and nature. The links are described along different dimensions. This first dimension focuses on the scientific aspect of heavy metal. It uses complex adaptive systems theory to show that the rapid diversification and evolution of heavy metal into multiple subgenres leads to a self-organizing and resilient socio-musicological system. The second dimension builds on the recent use of heavy metal as a critical thinking model and educational tool, emphasizing the artistic component of heavy metal and its potential to increase people’s awareness of environmental sustainability challenges. The relationships between metal, complexity theory and sustainability are first discussed independently to specifically show mechanistic links and the reciprocal potential to inform one domain (science) by the other (metal) within these dimensions. The paper concludes by highlighting that these dimensions entrain each other within a broader social-cultural-environmental system that cannot be explained simply by the sum of independent, individual dimensions. Such a unified view embraces the inherent complexity with which systems of people and nature interact. These lines of exploration suggest that the arts and the sciences form a logical partnership. Such a partnership might help in endeavors to envision, understand and cope with the broad ramifications of sustainability challenges in times of rapid social, cultural, and environmental change.

## Background

What does *complexity theory* (for definitions of terms in italics see Table 1) tell us about *heavy metal music* and its links to complex challenges as those present in global *environmental sustainability*? This question has been inspired by earlier scientific (Bachelard 1934, 1936) and artistic (Breton 1936) writings and recent calls (Angeler et al. 2016), which advocate the connection of isolated knowledge from different disciplines into a broader model of scientific inquiry. Specifically, this paper links the auditory arts, using heavy metal as an example, with two scientific disciplines, one comprising basic science (complexity theory) and the other applied research (environmental sustainability).

**Table 1:** Overview and definitions of terms used in this paper

Term	Definition
Complexity theory	Encompasses various approaches to the study of complex systems, including the application of complexity theory to strategy and organizations, the complexity of economics, self-organizing complex adaptive systems, chaos theory, theoretical computer science and mathematics and algorithmic information theory
Heavy metal music	A fast evolving form of the auditory arts belonging to the genre of rock music. Metal has roots in blues rock and psychedelic rock and originated in the late 1960s and early 1970s, mainly in the United Kingdom and the USA. Heavy metal has diversified into more than 20 subgenres with characteristic vocal, rhythm and instrumentation structure (see Table 2). This paper explores the scientific and artistic components of heavy metal to describe aspects of complex systems of humans and nature
Environmental sustainability/Sustainability Science	The interaction of human population with their environment in ways to guarantee their natural resources needs without compromising the ability of future generations to meet their needs. Sustainable development aims at avoiding overuse or degradation of resources to ensure long-term environmental quality
Complex adaptive systems	Hierarchical entities that consist of diverse and autonomous parts or components (“agents”) that depend on and relate with each other, and which are linked through many connections. Complex adaptive systems adapt to and learn from changes in the environment, which allows them to self-organize as a unified whole
Socio-musicological system	A particular from of complex adaptive system with reference to music, wherein system adaptation, learning and self-organization are governed by the broader and complex interaction between musicological, psychological, cultural, social, technological, marketing, and other economic aspects of social and technological change
Social-ecological system	A complex adaptive system of people and nature, where one component influences and is influenced by the other. For instance, the relationship between commercial fisheries, ecosystem service provisioning, resource overexploitation, aquatic resource degradation, loss of economic and social potential
Non-linear processes	Non-linearity manifests when not all agents in a complex adaptive system interact with the same strength which each other (e.g., species A and B in a system, or Y and Z, or A and Z, and so forth). Also when the loss of an agent in the system (e.g., a key predator in an ecosystem), can cause substantial change in biophysical interactions and a subsequent reconfiguration of the structure and functioning of the system
Delays	Delays in complex systems arise from the ability of systems to buffer the impact of disturbances. When the buffering capacity (mediated through feedback loops) is exhausted the delayed impact becomes manifest in structural and functional changes in the system. For instance, habitat degradation may make the persistence of large mammals untenable. However, the effects of degradation may not become evident until these mammals went extinct
Feedback loops	Circuits in complex systems that reinvest some of the yield to the input of a system to allow for self-correction and adjustment to internal and external variables
Balancing feedback loops	Also known as negative feedback loops. These occur when a changing initial condition lessens its change in the future. For instance, when the population number of predators increases, the consumption of their prey increases too, leading to a decrease in their population numbers. Due to increasing scarcity of prey, the population number of predators decreases as a result of the lack of food. Population dynamics of predator and prey are balanced
Reinforcing feedback loops	Also referred to as positive feedback loops. These occur when a changing initial condition furthers its change in the future. For instance, the greater the population of a species, the more progeny will be born. When those become adults, they will also have offspring. Rapid population growth rates of these species are reinforcing themselves and can ultimately have substantial impact on natural resources
Hierarchical organization	Refers to patterns and processes that occur at discrete scales of space and time. For instance, continental drift acts on millennial time scales and changes the face of the entire globe. By contrast, an annual grass is very short-lived and lives in a narrow space within a meadow
Information flow	Outcome of interaction between agents in a complex adaptive system that informs and influences the behavior and interaction of other agents in the system. For example, the collapse of the real-estate market in the USA in 2008 provided the “information” which “flew” across the world to affect the global economy at a systemic level
Connectedness	The degree and strength by which agents in a complex adaptive system interchange information based on their interactions. For instance, the economic crisis had a severer effect on southern European countries (strong connection with economic collapse) relative to northern European countries (weaker connection)
Regime shift	A regime shift occurs when a complex system changes from one set of structure, functions and processes to another set of structures, functions and processes. Regimes are stable and a system may not shift back to a previous regime without substantial and costly management

**Fig. 1:**
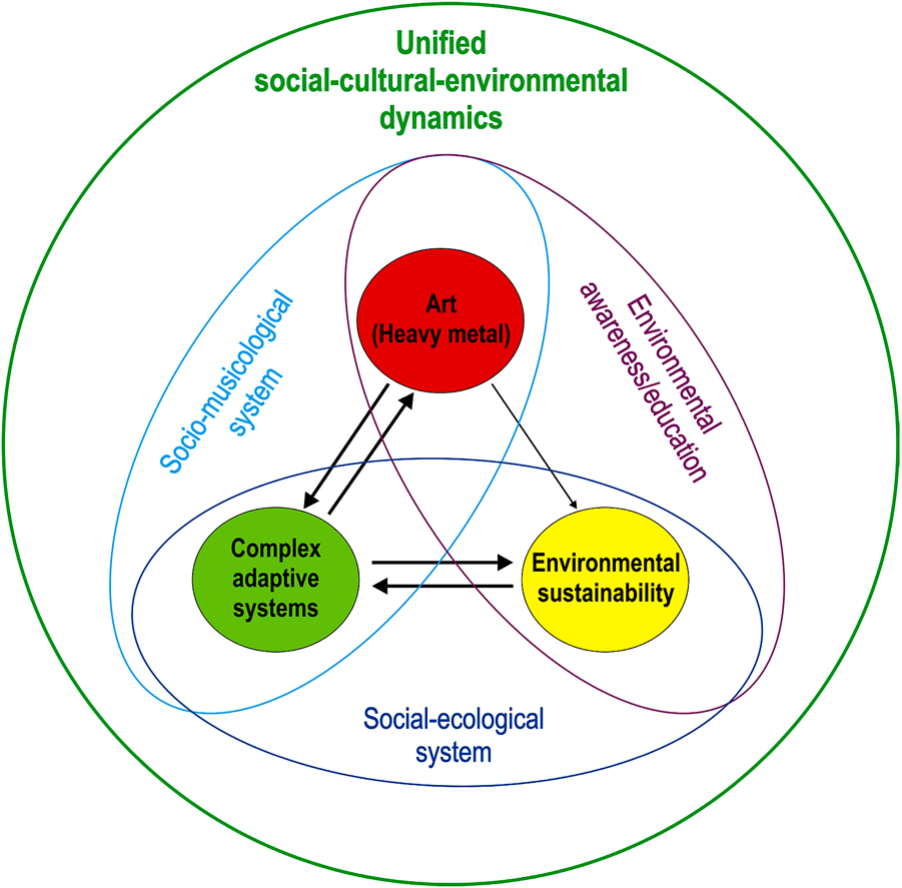
Schematic showing links between distinct, apparently unrelated knowledge domains. Shown are: (1) The links between heavy metal music and complex adaptive systems theory to describe the emergence of a socio-musicological system (*light blue ellipse*). (2) The potential of heavy metal music as an educational tool to increase awareness of environmental sustainability challenges (*purple ellipse*). (3) The links between complex adaptive systems and environmental sustainability that mediate social-ecological system dynamics (*dark blue ellipse*). (4) An emerging broader, unified picture of social-cultural-environmental dynamics (*green circle*). *Arrow directions* and *thickness* represent reciprocal versus unidirectional information potential between domains and interpretational objectivity versus subjectivity, respectively (see “Background” section)

The choice to link heavy metal with two apparently disparately distinct scientific disciplines is on purpose. Isolated knowledge from different disciplines can be linked in different ways and vary in their degree of subjectivity versus objectivity (Ratner 2002), the reciprocity by which one discipline informs the other (Rinia et al. 2002), or research method (qualitative and/or qualitative) used in transdisciplinary research (Angeler 2016). This variability is manifest in the approach used in this paper (Fig. 1), in which links between knowledge domains are made along three dimensions. The first dimension emphasizes the scientific component of heavy metal music (Brown 2011) in which it is linked to *complex adaptive systems* theory, a branch of complexity science, to describe the emergence of a *socio*-*musicological system* (Fig. 1). This paper will scrutinize the reciprocal potential to inform one domain by the other; that is, where heavy metal music potentially contributes to inform complex adaptive systems theory and vice versa (arrows in both direction in Fig. 1). Although this paper will only explore linkages theoretically, it is deemed possible that numerical analysis, which have been used in the study of complex adaptive systems (Holland 2014), might be used to study the heavy metal socio-musicological system. The potential to objectively study socio-musicological systems is shown by thick arrows in Fig. 1.

The second dimension builds on the recent use of heavy metal as a critical thinking model and educational tool (Angeler et al. 2016; Schmaltz 2016), emphasizing the artistic component of heavy metal (Kahn-Harris 2007) and its potential to increase people’s awareness of environmental sustainability challenges (Fig. 1). Information potential in this link is unidirectional; that is, heavy metal music informs only sustainability science. This unidirectional connection is made via allegories. These relate the music with specific environmental change challenges and ensuing human tensions with the aim to elicit thought and educate people about, and increase their awareness of environmental change. The thin arrow that symbolizes the unidirectional link (Fig. 1) means that the connection between heavy metal music and sustainability is entirely subjective. The unidirectional link also suggests that environmental sustainability issues currently do not influence metal music in ways to result in integral song architectures (lyrics, singing, rhythms, instrumentation) from which a “sustainability metal subgenre” might emanate. Figure 1 also shows a third dimension, in which complex adaptive systems theory and environmental sustainability are linked to describe the dynamics of complex systems of people and nature (*social*-*ecological systems*). The complexity of social-ecological systems dynamics has been reviewed recently (e.g., Allen et al. 2014) and will therefore not be explored here. However, this third dimension will be considered in the broader discussion of the synthesis between heavy metal, complex adaptive systems theory and environmental sustainability.

This paper will be structured in three parts. The first part emphasizes the scientific component of heavy metal music. It briefly sketches the claims of complex adaptive systems theory and then examines metal through the lens of this theory, including the description of operational terms to portray the emergence of a socio-musicological system. The second part of the paper focuses on the artistic component of the music and provides an overview of social-ecological systems challenges that are relevant from the perspective of environmental sustainability. It uses heavy metal songs in which their song architecture becomes allegorical to specific environmental challenges and potentially associated human tensions. These links are discussed within the context of environmental awareness and education. The paper concludes by synthesizing the linkages between the different knowledge domains in an overarching discussion of a broader and more integral understanding of social-cultural-environmental dynamics in times where planet Earth undergoes fast change of climatic, ecological, economic, production, communication and other technological systems (green circle in Fig. 1) (Folke et al. 2002; Berkes et al. 2003; Walker et al. 2004). This paper is intentionally descriptive to provoke thought and provide a source to stimulate future transdisciplinary research and cross-pollinate currently distinct disciplines.

## Heavy metal music: a complex adaptive system

Complex adaptive systems theory, a branch of complexity science, studies the systemic behavior of complex systems that results from the interaction of distinct features, including adaptation, recalibration and self-organization (Holland 2014). Complex adaptive systems dynamics have been described for a plethora of systems, including the cell, brain, and immune system of humans, ecological (social insect colonies, ecosystems, the biosphere), economic (global macroeconomic network, stock markets), technological (internet, artificial intelligence), political (geopolitical organizations, terrorist networks) and other social systems (schools, health services) (e.g., Meadows 2008; Holland 2014; Sundstrom et al. 2014). The use of complex adaptive systems theory for explaining the dynamic organization of disparately distinct systems suggests that an examination of metal as a complex adaptive system is worthwhile.

Heavy metal music, a fast evolving form of the auditory arts belonging to the genre of rock music that originated in the late 1960s and early 1970s, mainly in the United Kingdom and the USA, has been regarded in recent years as a cultural esthetic worthy of scientific study in its own right (Brown 2011). This is reflected in an increasing amount of scientific publications (Hickam 2014) since the first scholarly publications by Weinstein (1991) and Walser (1993). During the last 50 years, metal has diversified rapidly into more than 20 subgenres out of traditional or classic heavy metal (Sharpe-Young 2007; Table 2). Nowadays it spans a wide range of song architectures, lyric themes, and instrumentation, including distorted guitars, emphatic rhythms, dense bass-and-drum sound, and vigorous vocals, and the genre at large continues to develop fast (Brown 2011). This development has reached a complexity that is reminiscent of complex adaptive systems dynamics. That is, the development and diversification in metal has been the result of the broader and complex interaction between musicological, psychological, cultural, social, technological, marketing, and other economic aspects of social and technological change (Seppi and Stoycheva 2015), leading to a self-organizing socio-musicological system (Patel 2010).

**Table 2:** Overview of subgenres (including derivative forms) of heavy metal music and some of their characteristics (compiled and modified from sources on Wikipedia; https://en.wikipedia.org/wiki/Heavy_metal_subgenres)

Subgenres	Origin	Characteristics	Derivative styles
Alternative metal	Mid-1980s, USA	Has influences from alternative rock and genres not normally associated with metal; characterized by heavy guitar riffs, mostly melodic vocals and clean singing, unconventional song structures; uses sounds of other heavy metal genres; takes experimental approaches to heavy music	*Funk metal* (a fusion of alternative metal and funk) *Nu metal* (alternative metal blended with groove metal elements and other styles (e.g., grunge, industrial, funk and hip hop) *Rap metal* (institutes the vocal and lyrical form of hip hop; melodic singing and growling commonly associated with nu metal absent)
Avant-garde metal (avant-metal, experimental metal)	Mid-1980s, USA, Japan, Switzerland	Characterized by the use of innovative, avant-garde elements, large-scale experimentation, and the use of non-standard and unconventional sounds, instruments, song structures, playing styles, and vocal techniques. Evolutionary origins in progressive rock and extreme metal	
Black metal	Early to mid-1980s, Europe	Characterized by fast tempos, shrieking and/or growling vocal style, highly or heavily distorted guitars played with tremolo picking, raw (low fidelity) recording, unconventional song structures; artists often appear in corpse paint and adopt pseudonyms	*Ambient black metal* (fusion genre of either dark ambient or normal ambient music and black metal) *National socialist black metal* (NSBM; melds Neo-Nazi beliefs with hostility to some religions (Islam, Judaism); promote ethnic European paganism, occultism, or Satanism) *Red and anarchist black metal* (promotes Marxist, socialist, communist or anarchist ideology); was created as a reaction to NSBM) *Symphonic black metal* (incorporates symphonic and orchestral (strings, choirs, piano, organs, percussion, keyboards) elements) *Viking metal* (characterized by a focus on Norse mythology, Norse paganism, and the Viking Age; Nordic folk-influenced black metal; slow paced and heavy riffing style, anthemic choruses, uses clean and harsh vocals; frequent use of folk instrumentation use of keyboards for atmospheric effect) *War metal* (an aggressive, cacophonous and chaotic style of black metal)
Cello metal	1970s, United Kingdom	Characterized by the use of cellos and other bowed string instruments (violin, viola) as primary instruments, together with or replacing traditional rock instruments such as electric guitars, electric bass guitar, and drums	
Crust punk (Crust)	Mid-1980s, United Kingdom	Influenced by anarcho-punk, hardcore punk and extreme metal; songs with dark and pessimistic lyrics dwelling on political and social ills; sounds with strong bass component and distorted; often played at a fast tempo, occasional slow sections; grunting, growling and screaming vocals	
Death metal	Mid-1980s, USA	An extreme subgenre building on trash metal and early black metal; typically employing heavily distorted guitars, tremolo picking, deep growling vocals, blast beat drumming, minor keys or atonality, complex song structures, multiple tempo changes; often elaborates on the details of extreme acts, e.g. mutilation, dissection, torture, rape, cannibalism, and necrophilia	*Blackened death metal* (combines death metal and black metal) *Death ‘n’ roll* (inspired by and incorporates rock and roll elements to its overall sound) *Melodic death metal* (combines death metal with elements of the New Wave of British Heavy Metal, i.e. fast riffing and harmonic guitar lines) *Technical death metal* (characterized by fast, technically complex guitar and drum work, often including sweeping guitar solos)
Doom metal	Early to mid-1970s, USA, United Kingdom	An extreme form of heavy metal typically using slower tempos, low-tuned guitars and a much “thicker” or “heavier” sound than other metal genres; the music and the lyrics evoke a sense of despair, dread, and impending doom	*Death doom* (combines the slow tempos and pessimistic/depressive mood of doom metal with the deep growling vocals and double kick drumming of death metal) *Drone metal* (largely defined notes or chords that are sustained and repeated throughout a piece of music (drones); electric guitar is performed with large amounts of reverb and feedback; vocals, when present, are usually growled or screamed; songs often very long and lack beat or rhythm in the traditional sense) *Funeral doom* (blends death-doom with funeral dirge music; played at a very slow tempo, places emphasis on evoking emptiness and despair; electric guitars heavily distorted; keyboards or synthesizers used to create dark ambient aspects; vocals in the background and consist of mournful chants or growls) *Sludge metal* (blends elements of hardcore punk and southern rock; generally slow and heavy songs with brief hardcore passages; some bands emphasize fast tempos; guitars heavily distorted and producing an abrasive, sludgy sound; drumming often with hardcore d-beat or double-kick elements during faster passages; vocals usually shouted or screamed; lyrics generally pessimistic in nature)
Folk metal	Early to mid-1990s, Europe	Blends heavy metal with traditional folk music, including the widespread use of folk instruments and, to a lesser extent, traditional singing styles; sometimes features soft instrumentation influenced by folk rock	*Celtic metal* (fuses heavy metal and Celtic music) *Pirate metal* (blends metal music with classical pirate mythology, commonly combined with elements of Sea Shanties) *Medieval metal* (blends hard rock or heavy metal with medieval folk music)
Glam metal (hair metal, sleaze metal, pop metal)	Late 1970s, USA	Visual style of bands with artists styling their hair in teased-up fashion; combines elements of hard rock and heavy metal with punk rock and pop music, adding hooks and guitar riffs; borrows from the aesthetic of 1970s glam rock	
Gothic metal	Mid-1990s, Europe	Combines gothic rock with doom metal; lyrics generally melodramatic, fantasized, romantic, dark or sometimes gloomy; diverse range of vocal styles, including clean singing, growling and screaming, male and female singers	
Grind core	Mid-1980s, England	Fuses crust punk, hardcore punk, thrash metal and death metal; growling vocals, blast beats; very short songs (microsongs); very chaotic, lacks the standard use of time signatures; lyrics often focused on gore and violence, at times political	*Death grind* (death-grind or death/grind; fuses death metal and grindcore) *Pornogrind* (porno grind, porno-grind or pornogore; blends grindcore and death metal, using lyrics with sexual themes) *Goregrind* (characterized by its preoccupation with pitch-shifted or extremely low vocals; uses gore and forensic pathology as its exclusive subject matter; often very fast tempo)
Industrial metal	Mid-1980s, England, USA, Germany	Blends industrial dance music, thrash metal and hardcore punk; repeating metal guitar riffs, sampling, synthesizer or sequencer lines, distorted vocals	
Latin metal	Late-1970s, South America	Has Latin origins, influences, instrumentation, and Spanish vocals; Latin percussion and rhythm (e.g., Salsa rhythm)	
Metal core	Mid to late 1980s, USA	Combines heavy metal and hardcore punk; uses heavy guitar riffs and solos, drummers frequently use hardcore blast beats and double bass drums; vocal style includes death growls and shouting. A distinguishing characteristic is the “breakdown” (song is slowed to half-time and the guitarists play open strings to achieve the lowest-pitched sound)	*Melodic metal core* (combines sounds and traits from melodic death metal with hardcore punk, metalcore and at times emo; can have clean singing, growls and screaming; can feature harmonic guitar riffs, tremolo picking, double bass drums and metalcore-stylized breakdowns) *Deathcore* (combines death metal with metalcore or hardcore punk, or both; defined by an excessive use of death metal riffs, blast beats and use of hardcore punk breakdowns) *Mathcore* (not to be confused with math metal; rhythmically very complex with unusual time signatures; dissonant style of metalcore)
Neoclassical metal (Shred metal)	Late 1970s, America, Europe	Heavily influenced by classical music in its style of composition; uses a very technical style of guitar soloing called “shred guitar”, in which guitarists use cross-picking, sweep picking, and economy picking to play rapid scales and arpeggios; uses elements borrowed from classical music including instruments, scales and melodies	
New German hardness	Mid 1990s, Germany	A crossover style influenced by New German Wave, alternative metal and groove metal combined with elements from industrial, electronic and techno music; vocals (often in German) dominantly in deep, male, and clean voice; sometimes screaming and death growls	
Nintendo core	Early 2000, USA	Uses electric guitars, drum kits, and typical rock instrumentation; characterized by synthesizers, chiptunes, 8-bit sounds, electronically produced beats; stylistically very variable, including e.g. hardcore punk, post-hardcore, melodic metal core	
Post metal	Mid 1990s, Sweden	Similar to post-rock, but tends to include lower-tuned and distorted guitar(s), heavy atmospherics, gradual evolution of song structure to a crescendo or climax (or multiple ones within a song); minimal emphasis on vocals; often instrumental; lyrics frequently abstract (thematic or philosophical)	
Power metal	Mid 1980s, Germany, Scandinavia, USA	Takes influence from heavy metal and speed metal; often emphasizes clean, melodic, high-pitched vocals, fast pacing (double bass drumming), and melodic lead guitar; rhythm guitar defined by straight power chord progressions; harsh vocals used at times (backing vocals); lyrics based on fantasy themes; generally more upbeat than other metal genres, seeking to empower the listener and inspire joy and courage	
Progressive metal	Mid 1980s, Australia, United Kingdom, North America	Fusion between progressive rock and heavy metal; complex structure with unusual and dynamic time signatures, long compositions, skilled instrumental playing; vocals, if present, are melodic (at times unclean); lyrics often philosophical, spiritual, or political	*Djent* (characterized by progressive, rhythmic, and technical complexity; features heavily distorted, palm-muted guitar chords, syncopated riffs and polyrhythms alongside virtuoso soloing)
Speed metal	Late 1970s, Europe, America	Extremely fast, abrasive, and technically demanding; usually less abrasive and more melodic than thrash metal; less influence from hardcore punk; faster and more aggressive than traditional heavy metal; inclination to virtuoso soloing and featuring short instrumental passages between couplets; uses highly expressive vocals, but lesser use of harsh vocals than in thrash metal	
Stoner metal (Stoner rock, desert rock)	Early 1990s, California	Combines elements of heavy metal, psychedelic rock, blues rock, acid rock, and doom metal; typically slow-to-mid tempo and features a heavily distorted, groove laden bass-heavy sound, melodic vocals, and “retro” production	
Symphonic metal	Mid-late 1990s, Europe	Influenced by early gothic metal, power metal and symphonic rock; includes elements of classical music (symphonic instruments, choirs, full symphony orchestra); keyboards often find a dominant place; classically trained female vocalists and a second vocalist performing growls are common	
Trash metal	Early 1980s, USA, Europe	Extreme subgenre with fast tempo and overall aggression; songs usually with fast percussive beats and low-register guitar riffs overlaid with shredding-style lead work; lyrics often deal with social issues using direct and denunciatory language; approach partially overlaps with the hardcore genre	
Classic (traditional) heavy metal	Late 1960s, USA, UK	Seminal genre from which today’s subgenres evolved and diversified; departs from the original blues roots of hard rock; characterized by mid-to-fast-tempo riffs, thumping basslines, crunchy riffs, extended lead guitar solos; vocals clean, often high-pitched, anthemic choruses; pioneering use of double lead guitar	
*Umbrella terms*			
Christian metal	Late 1970s, USA, Sweden	An ideological umbrella form of heavy metal that comprises almost every subgenre of heavy metal music; defined by dedication to and using song lyrics based on Judeo-Christian traditions. Unblack metal (aka Christian black metal) refers to stylistically black metal which promotes Christianity in their lyrics and imagery)	
Dark metal		A loosely defined subgenre of heavy metal, with stylistic origins in gothic and extreme metal	
Extreme metal	Early 1970s, Europe, USA	Comprises a number of related heavy metal subgenres with a more abrasive, harsher, underground, non-commercialized style or sound associated with the thrash metal, black metal, death metal and doom metal genres, and sometimes speed metal. Despite being non-mainstream music, extreme metal has influenced an array of musical performers inside and outside of heavy metal	
Pagan metal	Early 1990s, Northern Europe	Fuses extreme metal with the pre-Christian traditions of a specific culture or region; uses specific thematic concepts, rustic melodies, unusual instruments or archaic languages; often associated with Viking metal and folk metal	

Umbrella terms that encompass several genres are also shown. Note: This classification is subjective and at times incomplete but serves as a summarizing construct to show complex adaptive system dynamics in heavy metal. Given the subjectivity, there can be disagreement among artists, fans, and critics about membership of selected bands to these subgenres (Tsatsishvili 2011). This paper therefore refrains from giving examples to avoid bias

Meadows (2008) and Holland (2014) give simple overviews of complex adaptive systems, and provide the basic criteria for a relatively uncomplicated examination of metal through the lens of complexity science. My examination of metal as a complex adaptive system is based on a theoretical model because of two reasons. First, metal is a very young field with many gaps in the scientific literature, and it currently lacks the necessary broader peer-reviewed quantitative and qualitative information to populate/parameterize models empirically and realistically. Second, even if this knowledge was available, model predictions are highly uncertain because, as noted by Holland (2014), a full description/understanding of complex adaptive systems is impossible. There is, for instance, emergent behavior that cannot be described by the sum of individual components, which often results from *non*-*linear processes*, *delays*, and complex *feedback loops* (see descriptions and examples in Table 1). However, theoretical models have value in building a foundation for future research (Meadows 2008).

**Fig. 2:**
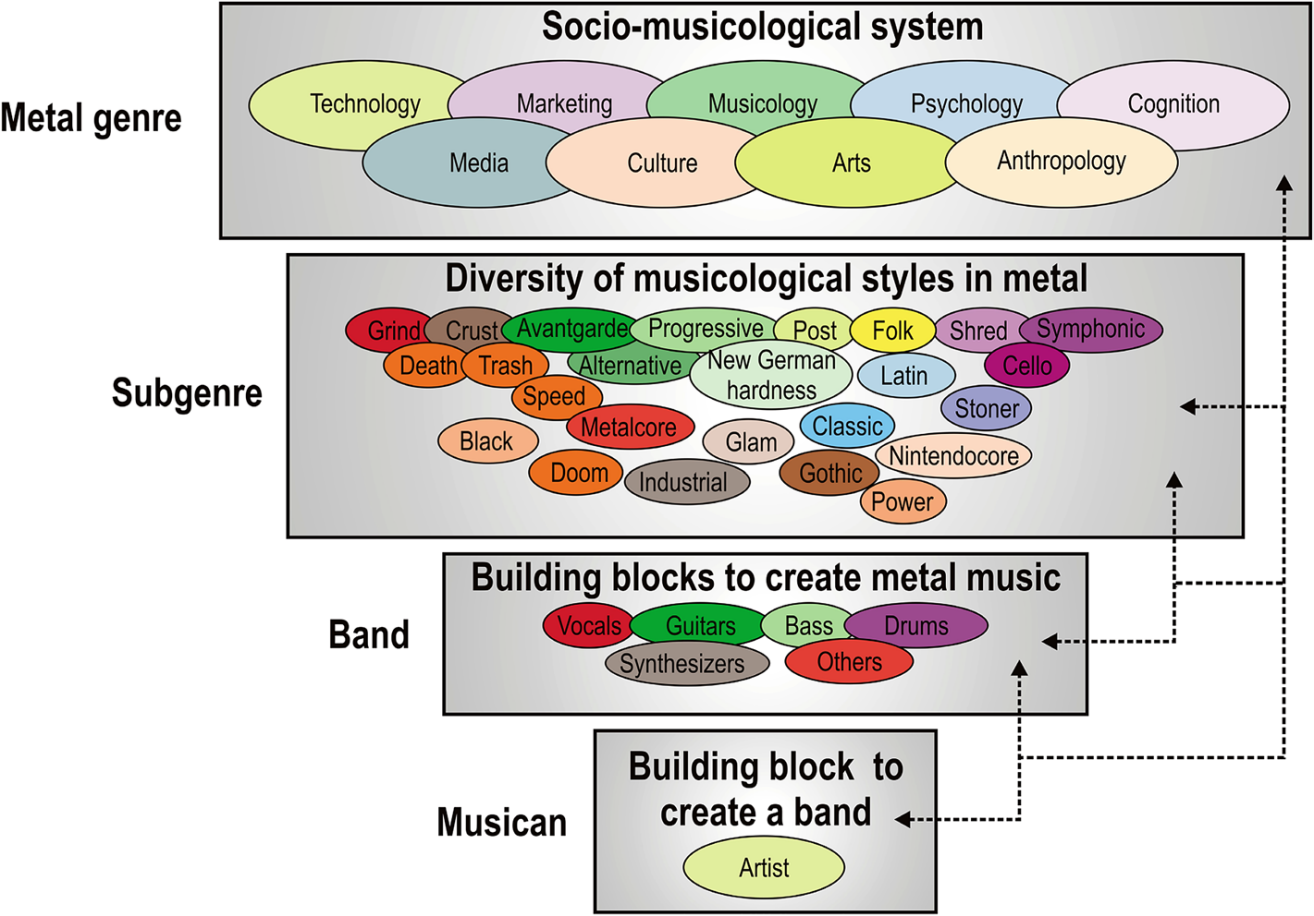
Simplified complex adaptive system model of heavy metal music showing discrete hierarchical structure and the connections between those. Non-linear interactions (connectivity between agents) are symbolized by *different colors*. These *colors* are not an absolute representation of dynamic interactions and only serve to visualize non-linear system connectivity; interactions and similarities are therefore not an absolute representation of dynamic interactions in the heavy metal complex adaptive system

My theoretical model of the metal socio-musicological system is based on the description of the general elements that make up a complex adaptive system (Fig. 2). Basic elements of a complex adaptive system are: *hierarchical organization*, *information flow* within and between these hierarchies and other complex systems (i.e., *connectedness*), non-linear patterns and delays resulting from information flow, and the deriving *reinforcing* and *balancing feedback loops* (Meadows 2008; Holland 2014). These feedback loops comprise the manifold processes that drive system behavior, and help the system recalibrate and adapt to disturbance. From these basic system elements emerge innovation and learning, resilience, self-organization and system-level persistence.

### Hierarchical organization

The hierarchical organization of the metal complex adaptive system is based, from a structural point of view, on a series of “actors” and begins with the musician at the lowest operational level. Lowest operational level means that there are more levels down the hierarchy; that is, musicians as humans are built up of organs, cells, organelles, molecules and atoms but these levels are likely marginal for the understanding of the metal complex adaptive system. The structure of the complex adaptive system increases in complexity with increasing hierarchical level, which is reflected in an increasing number of actors. That is, the next level up the operational hierarchy of the metal complex adaptive system is the band comprising various musicians. Various bands playing similar musical styles collectively comprise the subgenre, the next level in the hierarchy (Table 2; Fig. 2). The highest operational level is the genre of heavy metal music. This level is not only composed of the plethora of musicians and bands across the multiple subgenres but also other actors from distinct social environments that interact with each other. These include fans, music producers, technologists, scientists from different fields (e.g., culture and art studies, anthropology, psychology), marketing professionals, and politicians. Combined, this broader community of different actors makes up the socio-musicological system that comprises the metal complex adaptive system. There are further levels up the hierarchy (e.g., the whole biosphere); however, these may go beyond the upper boundaries necessary for describing the structure of agents and thus the highest operational hierarchies of the metal complex adaptive system. This does not mean, however, that there aren’t functional linkages (some of these will be explored in the Synthesis section). That is, if the whole biosphere undergoes significant biophysical change it is likely that it will affect all the levels subsumed within it.

### Information flow

*Information flow* and *connectedness* among the structural agents in complex adaptive systems is critical for system function and behavior. Connectedness reflects the structural linkages between agents in the complex adaptive system; these linkages can vary in strength and connect some agents more and others less in the system. For instance, musicians in a band are closer connected to each other than with the musicians of another band. Information flow reflects the functional linkages between the connected agents. That is, the “information” created from the vocalists, guitarists and drummers “flows” between the musicians and leads to the emergence of a song. This example shows that information flow in complex adaptive systems is goal oriented. Specifically, in the metal complex adaptive system it is the generation of a specific form of music that often reflects transgressive cultural and political norms and values (Kahn-Harris 2007; Brown 2011). These norms and values often build on the incorporation of pornographic and forensic themes in their lyrics (e.g., pornogrind, grindcore; Table 2). To this end, agents need to be connected within and across hierarchical levels (Allen et al. 2014), but these connections follow certain rules and vary in strength. In the metal complex adaptive system, individual musicians either act as front man, play the guitar, bass, drums, occasionally synthesizers, and sometimes subgenre specific instruments (Table 2). The rule is that different combinations of these musicians are needed to qualify as a metal band that generates that specific style of music (metal; not classical music). A choir composed of only singers does not qualify as a metal band that plays metal music.

At the band level of the metal hierarchy, the information flow, which comprises the distinctive and characteristic series of sound structure that emerges from the combination of vocals and instruments, is manifested in a song architecture that characterizes the band’s label. For instance, doom metal bands typically use slower tempos, low-tuned guitars, and “thicker” or “heavier” sound than other metal subgenres, and the music and lyrics evoke a sense of despair, dread, and impending doom. In metalcore and deathcore, a harsher version of metalcore, a distinguishing characteristic is the breakdown, whereby the song is slowed and the guitarists play open strings to achieve the lowest-pitched sound. Folk metal blends heavy metal with traditional folk music, including the widespread use of folk instruments and, to a lesser extent, traditional singing styles. These examples show the higher within-subgenre similarity rather than between-subgenre similarity of band styles, and more broadly how subgenres are connected. Metalcore and deathcore with their breakdowns are more similar to each other than to death metal and trash metal without breakdowns. However, when compared with symphonic metal and classic metal, which are more melodic subgenres, the former metal subgenres become more similar to each other with their overall harshness and aggression. Sometimes metal subgenres are so similar to each other that it is difficult to draw boundaries (Tsatsishvili 2011). As a result, some umbrella terms have been defined that unite similar metal subgenres (Table 2).

That agents in a complex adaptive system are linked and information flows with different strengths among agents reflects another critical hallmark of complex adaptive systems, their nonlinear patterns and processes. Such *non*-*linearity* can be seen in different patterns of connection of elements of the metal complex adaptive system. For instance, from a scientific viewpoint, many papers dealing with metal have been published in psychology, meaning that there is high information flow and connectedness between metal and psychology (Brown 2011). To my knowledge none exists between the music and complexity and sustainability science, highlighting the lack of connectedness and information flow between these domains. There is no linear relationship when comparing connectedness in these examples. Similarly, from a cultural-political perspective, Western music is more connected to metal than middle Eastern cultures, in which this form of music doesn´t meld well with cultural, religious and ethical belief models (Stanton et al. 2012). Metal is emerging in countries in Asia and Latin America (Wallach 2005; Varas-Díaz et al. 2014), and this emerging link suggests an increasing degree of connectedness of these countries with metal.

Connectedness also has a temporal, dynamic dimension, operating at different speeds across hierarchical levels in the metal complex adaptive system. A metal song typically lasts from seconds to minutes. A subgenre needs years to develop. Metal has now evolved over the past 5 decades. These temporal patterns are also nonlinear, and can comprise a feature characteristic of complex adaptive system dynamics: delays (Table 1). The different speeds also have implications when delays can be perceived. A delay in a complex adaptive system becomes evident when accumulated structural and functional changes in the system suddenly become evident (Table 1). At the song level, such delays are often manifested in unusual time signatures, resulting from unrhythmic patterns when sound elements are synchronized or unsynchronized with lags. This is a characteristic of mathcore, grindcore, and progressive metal. In songs from these subgenres rhythmic delays can be perceived immediately. Other metal subgenres (symphonic metal, power metal) are more melodic and are therefore characterized by fewer rhythmic delays.

At the other extreme, at the highest operational level, the perception of delays might not be easy because current system dynamics operate so slowly that their effects eventually manifest in the future, as is the case with substantial biophysical change in social-ecological systems (Folke et al. 2004). Metal is currently seeing transforming social perceptions and music industries. Music digitalization, rapid popularization of online streaming services (YouTube, Spotify, Facebook, SoundCloud), subscription models, and social media websites facilitate broad exposure. Quickly expanding regional and global metal markets, festivals, and conferences enable social engagement worldwide (Seppi and Stoycheva 2015). In this regard, metal shows a similar “teleconnection” as social-ecological systems (Adger et al. 2009). That is, geographically disparate regions are becoming increasingly connected and one region may affect another remote area due to this global connectivity (e.g., metal styles that have emerged in Sweden [djent] may inspire metal in the USA and elsewhere). In social-ecological systems, for example the production of food and fiber shows similar teleconnection (Rist et al. 2014).

It is clear that the globalization of metal, and music in general, can lead to novel interactions. A case in point is the interaction of metal with other musical genres or sounds from emergent technologies. Nintendocore exemplifies how chiptune elements and other sounds from video gaming (a technological invention) can entrain heavy metal music, leading to a new subgenre. Alternative metal, especially its crossover forms rap metal and nu metal, incorporate elements from hip hop music, a form of contemporary music characterized by rapping (chanted rhythmic and rhyming speech). On the other hand, symphonic metal and cello metal emphasize a strong classical component, particularly in terms of instrumentation used.

Metal is influenced by other genres, but also affects other musical genres. For instance, certain metal elements are reflected in brostep, an emergent form of dubstep, which is a genre of electronic dance music. These interactions between metal subgenres and other music forms and technological inventions exemplify a vast potential and source of dynamic interactions between complex adaptive systems (Kahn-Harris 2007; Brown 2011). These dynamic interactions often follow from the constant recalibration of a complex adaptive system in response to a changing environment. However, the outcomes of these dynamics are currently highly uncertain, partly because of delayed responses that might arise from these interactions. Delays, or time lags, also make predictions of when and why systems are changing extremely challenging.

Uncertainty, highlighted in the above examples, is a general phenomenon in complex systems dynamics. It stems from the stability of current patterns and processes in a resilient complex adaptive system. Resilience refers to the ability of a system to maintain structures and functions in the face of disturbances, and when this capacity is exceeded systems reorganize into a new regime with different structures and functions (Holling 1973). Critical to the understanding of resilience are feedbacks, or feedback loops, that arise from the flow of information and connectivity in the complex adaptive system.

### Feedback loops

As is the case with many complex adaptive systems (Sterman 1994), there are potentially far more feedbacks in the metal socio-musicological system than can be currently envisioned and enumerated in this paper. However, some examples shall help make the point that feedback loops essentially contribute to complex adaptive system dynamics in metal.

There are *reinforcing* and *balancing* feedback loops, and these can operate within and across hierarchical levels in the complex adaptive system and affect processes from both directions in the loop (Meadows 2008). Imagine at the band level agreement among musicians and similar opinions about music style and where to perform concerts. Based on these criteria, bands compose new songs and make a new release or concert. After each release or gig the band composes new songs and releases a new album and presents it at another festival. The band works in a balancing feedback loop, whereby the generation of new music is partly contingent on the music created before. In this calibration process, the musicians and the band at large learn, and this learning process builds the source for future experimentation and innovation. A reinforcing feedback loop becomes evident when there is disagreement and escalating conflict between band members; that is, the reinforcing feedback loop results in an initial conflict that increases over time to further even more conflict in the future. Less cooperation can lead to less songs being produced and consequently fewer releases and/or concerts. With increasing escalation of conflict learning, experimentation and innovation is de-emphasized, contributing to a downward spiral that can culminate in the band´s dissolution. There can be of course other factors (personal, professional) leading to the disappearance of a band.

Balancing and reinforcing feedbacks at the band level may scale up to the next hierarchy, the subgenre level of metal. When balancing feedback loops dominate over reinforcing feedback loops, the whole subgenre is likely to persist and further develop. If the relationship is inverse, a subgenre likely disappears. Balancing and reinforcing feedback loops have influenced metal subgenres distinctly. For example, many subgenres see a rapid diversification into derivative styles, based on the constant recalibration resulting from learning that balances feedback loops. For instance, alternative metal diversified into nu metal and funk metal, and death doom, drone metal, funeral doom, and sludge metal derived from doom metal (Table 2). The New Wave of British Heavy Metal that peaked between 1975 and 1985 and which has seen posterior reformation exemplifies that feedback loops can change over time.

Broader social-political environments can exemplify feedback loops at the highest operational hierarchies. Western countries do not generally impose restrictions on cultural, artistic, and scientific development within permitted legal settings. This contributes to and facilitates recalibration, innovation and progress across social environments, and ultimately systemic evolution. As stated before, there are many feedbacks operating between the constantly developing scientific, musicological, economic and marketing aspects in the metal socio-musicological system that ultimately allows it to evolve. On the other hand, in many countries with conservative politics or specific belief models there are more restrictions to development. Progress is often limited due to suppressive and penalizing action that may reinforce incompatibilities between cultural models. Such incompatibilities can be reinforced to such an extent as to lead to violent action, as witnessed during the recent terror attacks at a metal concert in Paris. So too, is the use of heavy metal (and children´s TV programs Barney and Sesame Street) in psychological operations used in a negative context, for instance during the interrogation of detainees (BBC 2003) that can reinforce hard feelings towards metal. These examples highlight that top hierarchies are connected to and influence behavior at lower levels.

In short, metal has seen an expansion and gain in world-wide popularity (Trilling 2007), despite some periods of turmoil in the 1990s that forced musicians to distance themselves from the genre, due to a plethora of factors including aversion by the media and music industry (Konow 2002). Despite this, metal has been resilient to this disturbance. It shows that balancing feedback loops have dominated over reinforcing feedback loops thus far in the evolution of metal.

In summary, the broader dynamics of metal abides within a dynamic state space that adapts and flexes constantly based on the complex interaction of agents in the system (Lansing 2003; Frenken 2006), dynamics which can be seen in art at large (Levine and Levine 2011). Social theory on diffusion of art forms shows that some elements climb up and thrive in this state space, and others fizzle out as dead ends based on the structure of social interactions (reminiscent of the viral dynamics of marketing; Leskovec et al. 2007). The state space of the metal socio-musicological system comprises similar complex dynamics driven by endogenous and exogenous factors (social perception, changes in music production and routes of exposure, human behavioral, cognitive, and cultural aspects) and interactions. Ultimately, these dynamics highlight the ability of the metal complex adaptive system to persist in broader social and cultural environments. It has become a self-organizing, self-maintaining, resilient socio-musicological system.

## Metal and environmental sustainability

The previous section emphasized the scientific component of heavy metal music (Brown 2011) and explored its merger with complexity science to describe the emergence of a complex socio-musicological system. This section uses an independent line of inquiry. It emphasizes the artistic component of heavy metal and explores its use as an educational tool to increase people’s awareness about environmental sustainability challenges on planet Earth that undergoes fast change in climate, ecological, economic, production, communication and other technological systems (i.e. global change) (Folke et al. 2002; Berkes et al. 2003; Walker et al. 2004). The use of metal music as a critical thinking and educational tool has been recently highlighted (Angeler et al. 2016, Schmaltz 2016).

*Sustainability science* is concerned with the ability of systems of people and nature to adapt to changing social-environmental baselines (Kates et al. 2001). Natural disasters (floods, wildland fires, hurricanes, infectious diseases), terrorism, or mass migrations of people due to humanitarian crises have increasingly shattered the world in recent years. In its extreme form, the entire globe can undergo a fundamental reorganization because of the broader overexploitation of resources and climate change (Rockström et al. 2009; Hughes et al. 2013). There is much uncertainty about global change outcomes, but negative consequences (conflicts, health problems) and associated human tensions (distress, aggression, depression, torture, despair) are and will likely become a more common denominator in people´s lifes in the future (Horner-Dixon 1991; McMichael et al. 2008). Sustainability science is in critical need of qualitative and quantitative approaches to address these and many more consequences that result from global change, to facilitate citizens’ comprehension of the scientific and potential policy dimensions of environmental change, especially because it is the general public that bears the costs of transformation and adaptation measures (Angeler 2016).

Here, metal is explored as one qualitative tool that provides a critical thinking and education model about such complex challenges. Artists and scientists are increasingly collaborating (e.g., EcoArt movement) to communicate the nature of problems, heighten awareness of ecological concerns, search for new solutions, and design ecological activity to enable public action (Kagan 2014). A series of approaches is used to communicate environmental change problems, ranging from realistic (e.g. photography) to abstract, often aiming to elicit human tensions (Peeples 2011). Shock tactics are often used to reinforce the surprise component and stimulate people´s learning and long-term memory formation (Timms 2004, Lisman and Grace 2005). With regards to art, and similar to the Dada movement in the early twentieth century, it has been argued that forms of heavy metal have systematically transgressed the boundaries of the accepted in the auditory arts (Kahn-Harris 2007), and other music forms (e.g., post-punk, New Complexity School) might similarly deviate from these boundaries. This suggests that metal can symbolize artistically when change potentially takes people out of comfort zones.

Although not unique to metal music alone, individual subgenres of metal (Table 2) have song structures that become allegorical to and can symbolize specific aspects of human tensions associated with disturbances in social-ecological systems (e.g., natural disasters, terrorist attacks, humanitarian crises; see below). Songs from these subgenres can be linked with these specific social-ecological system challenges artistically, although these specific links are entirely subjective, as many artistic expressions are in general. I showcase this potential with selected examples (Table 3) from a potentially vast spectrum of application. Note that band and song names are of subordinate importance in symbolizing these sentiments. That is, although the songs were not always written to intentionally make the connection to environmental sustainability challenges, it is possible to draw a comparison between the sounds and lyrics the music inspires in many people´s emotions and sustainability challenges. In the examples below, emphasis is placed on contextualizing metal songs with specific environmental change challenges from which emotional reactions in people possibly ensue. The metal allegories comprise an approach, based on the visual arts (Thomsen 2015), which has potential to reconceptualize “hearing” as “questioning”. That is, the allegorical expression of environmental change issues through metal might trigger a chain of questioning: for instance, what is environmental change and why do we need to study it? How do these challenges relate to sustainability? What are elements of uncertainty and surprise, and how do they manifest? This process can improve learning and connect people more closely with environmental sustainability issues (e.g., Ryan 2001; Angeler 2016). A combination of metal with visual underpinnings (e.g., photography; Thomsen 2015) may further bolster people´s connection to environmental change problems. Also, there is precedence in the use of metal sound tracks to provide auditory support in war (Black Hawk Down), horror (Valentine), action/science fiction (The Matrix), or fantasy (The Crow) movies and film series (Resident Evil). O’Brien (2012) deals specifically with the use of metal in combat movies.

**Table 3:** Selected sustainability challenges in social-ecological systems (SES) and how they manifest in the form of heavy metal allegories

SES challenge	Emotions in people	Manifestation in metal allegory	Song (Band/artist) examples	Links to YouTube^a^
*Ecological and social disturbances*				
(a) Before disturbances	Widespread positive emotions; ignorant of potential catastrophes in immediate environment	Melodic aspects in symphonic metal; joy and empowerment conveyed in power metal	Escapist (Nightwish) March of time (Halloween) Evoke (After Forever) Angels (Within Temptation)	https://www.youtube.com/watch?v=ybyU0pdIu4Y https://www.youtube.com/watch?v=3sQ5iaynO8k https://www.youtube.com/watch?v=N7BFBW3psp0 https://www.youtube.com/watch?v=4ifTjdKrTPw
	Sense of false security	Psychedelic elements in stoner metal	Dopethrone (Electric Wizzard)	https://www.youtube.com/watch?v=Gz-xmICHCK4
(b) During disturbances	Chaos, uncertainty	Rhythmic complexity and unpredictability in mathcore and grindcore	Concubine (Converge) Garrucha (Car Bomb) Agorapocalypse now (Agoraphobic Nosebleed)	https://www.youtube.com/watch?v=gIB9Cai5kZ4 https://www.youtube.com/watch?v=HjFdhraTA7A https://www.youtube.com/watch?v=Z3vkeO-GsV8
	Overwhelming exposure to and lack of control of intense and fast disturbances (e.g. floods)	Fast tempo and aggression in speed metal and trash metal	Unanswered (Suicide Silence) Speed metal messiah (Joe Stump)	https://www.youtube.com/watch?v=VT13aP_GnuM https://www.youtube.com/watch?v=KxQZsW0H7LU
(c) Aftermath of disturbances	Widespread negative emotions; despair, agony, stress	Despair conveyed in doom metal; death reflected	Murdered by grief (Frowning) Among the falling stones (Ophis) Morte aetérna (Chelsea Grin)	https://www.youtube.com/watch?v=VSSFG_pNPg0 https://www.youtube.com/watch?v=jLE8FCn6BzE https://www.youtube.com/watch?v=P1LXARJv4Lg
(d) Social and political causes	Dissatisfaction against governments	Crust punk lyrics dwelling on social/political ills	Dig their own graves (Corrupt Leaders) Fucking bastards (Behind Enemy Lines)	https://www.youtube.com/watch?v=eSGEKQaaRjo https://www.youtube.com/watch?v=-N7IVV6yIS0
*Systemic SES aspects*				
(a) Regime shifts	Often not perceivable given long time scales, but metal can serve as model to conscientize people	Transitions in songs in the form of breakdowns in metalcore and deathcore	Resistance (Veil of Maya) In Sincerity (For the Fallen Dream) Neo Soul (After the Burial)	https://www.youtube.com/watch?v=Vd3IT1LuHfM https://www.youtube.com/watch?v=_JH-dmHtZ4M https://www.youtube.com/watch?v=9hOghDTPvJg
(b) Undesired SES states (poverty traps)	For instance, health and disease related emotions	Abrasiveness and harshness in black metal	Demonium (Inmortal) Intent to kill (Dawn of Demise) A.E.O.N. (Sybreed)	https://www.youtube.com/watch?v=fk83pC0xDeo https://www.youtube.com/watch?v=VDke1Gvmr3s https://www.youtube.com/watch?v=HPe9Uv8gHnw

Examples with online links to YouTube are given. Note that several metal allegories apply to different SES challenges highlighting their broader utility for symbolizing sustainability issues

^a^Some of the videos are preceded by advertisements that are unrelated to the songs on YouTube

### Metal, human tensions and environmental sustainability

Disturbances as manifestations of social-ecological system dynamics can be associated with a variety of metal subgenres that are allegorical to human emotions. That is, these subgenres have the necessary diversity to characterize allegorically distinct sustainability issues and experiences and sentiments of people prior, during and after disturbances.

In a social-ecological context, imagine a society that lives in a peaceful environment, urban or rural, where their basic requirements for livelihood are covered, including recreational and other services provided by nature. People live with a sense of relative security and happiness. These conditions and sentiments can be expressed with the more melodic symphonic metal subgenre or power metal that seeks to empower the listener and inspire joy and courage. However, the sense of security can be false when people take their comfort for granted. Occasionally sludge metal, and stoner metal with its psychedelic elements, which reflects a temporary altered state of consciousness induced by the consumption of psychedelic drugs, can symbolize feelings of false reality or security as catastrophes can happen at any time.

With climate change, the frequency, duration and magnitude of disturbances are altered. For instance, devastating wildfires or extreme precipitation events that result in flashfloods occur more often in a changing climate (Christensen and Christensen 2003; Gillett et al. 2004). The devastation of torrential floods or wildfires occurs relatively fast, causing death and destroying the basis of people’s livelihoods. Metal genres with fast tempo and overall aggression in sound and vocals, like speed metal or trash metal, can symbolize the fast devastation when floods or fires unleash and peoples reactions to these catastrophes. People´s despair, agony and suffering in the aftermath of these disturbances becomes allegorical to many sentiments that doom metal inspires with slower tempos, low-tuned guitars and thick/heavy sounds.

Similar sentiments can be expressed with social disturbances. The world currently witnesses large scale human migrations caused by war. The sense of agony of people fleeing their countries with an associated loss in economic and social potential, culminating in humanitarian catastrophe, can be brought to mind with the sentiments death doom and funeral doom inspire. Crust punk and grindcore, which consciously emphasize political and social ills, can symbolize the sources of conflict that can result from corrupted and/or authoritarian governance regimes.

Chaos and uncertainty are high during and immediately after disturbances, resulting in discomfort from not knowing how the disturbance will end or what the future holds. Uncertainty and chaos may be associated with the patterns of mathcore’s, grindcore’s, and progressive metal´s rhythmic complexity. These subgenres are characterized by unusual time signatures, atonality and dissonance in the manifestation of song elements that are reminiscent of chaos. The listener is constantly challenged to digest and anticipate dynamically interacting and often antithetical sound patterns and rhythm structures. These metal subgenres, along with other types of media and art, have therefore high potential to increase people´s awareness about the sentiments and behavior of uncertainty and chaos that people may experience as a result of natural disasters.

Similar applications of metal allegories can be used in other contexts, highlighting their broader use to symbolize disturbances in social-ecological systems. There is a wide range of social issues that lend themselves to the emotional content of metal, such as the stresses particular to urban living for instance, recurring overarching problems in public transportation during rush hours, attacks in schools, or broad-scale terrorist massacres in urban public places.

In social-ecological systems disturbances can ultimately lead to wider systemic changes resulting in substantial reorganization of the system’s structure, function and dynamics (i.e., regime shifts). *Regime shifts* occur when for example a shallow lake with clear water and submerged vegetation shifts into a nutrient-enriched, turbid-water regime with frequent algal bloom outbreaks. This nutrient-enriched regime is stable, meaning that the lake will not return to a clear-water regime without substantial management. Such systemic changes of social-ecological systems can be explored through metal songs from distinct subgenres. Regime shifts per se that characterize the non-linear transitions between system regimes can be characterized with metalcore and deathcore songs. These songs symbolize the dynamism inherent in non-linear transitions in the form of “breakdowns” that introduce sudden changes in beat and rhythm patterns in a song; that is, breakdowns in metalcore and deathcore is figurative of the existence of and can inspire people about dynamic changes between alternative regimes in social-ecological systems. A further systemic aspect of social-ecological system change becomes evident when systems have shifted and locked in a new regime after a disturbance threshold has been passed. Similarly to the above examples of sentiments people may experience in the aftermath of a disturbance, metal allegories based on more abrasive and harsher black metal, can be used to characterize the persistence of an undesired post-disturbance regime; for instance, when a system locks in poverty traps, which are characterized by limited access to credit and capital markets, extreme environmental degradation (which depletes agricultural production potential), corrupt governance, capital flight, poor education systems, disease ecology, lack of public health care, war and poor infrastructure (Bonds et al. 2010). To demonstrate such cases, metal can be used to increase people´s awareness that systems are often not able to rebound from disturbances, but rather become trapped in an undesirable regime for humans, and which are very difficult to escape (e.g., Cinner et al. 2009).

## Synthesis

The preceding sections explored hitherto nonexistent links between metal, an auditory art, complex adaptive systems and environmental sustainability science along two independent dimensions; that is, from a socio-musicological systems point of view and an environmental education/awareness perspective (Fig. 1). These lines of inquiry show that metal as an art form has potential to describe distinct and independent facets of the complexity of systems of people and nature. The paper concludes with an examination of the overarching implications that may derive from the merger of these independent explorations.

There are many different complex adaptive systems in systems of people and nature (Sundstrom et al. 2014). Arguably, art comprises a complex system that is embedded in a broader social-cultural-environmental complex adaptive system (Fig. 1). Within the arts there are many forms and genres that might be nested and interacting complex adaptive systems. Using heavy metal as an example, this paper shows how a musical form can become its own complex adaptive system or socio-musicological systems. Such systems are often artificially separated in scientific research, which impedes the development of appropriate models and knowledge of system dynamics. The emergence of metal not only reflects cultural change in modern societies, its dynamics follow those described for complex adaptive systems. Metal thus provides a valuable addition to complex adaptive systems research and further highlights that considering the arts as a form of complex adaptive system is long overdue. The bottom line is that it is uninformative to consider the arts and sciences as discrete units (Friedenwald-Fishman 2011). The arts and the basic and applied dimensions of science are integral to humanity. People cannot be understood as separate from our environment, be it ecological, cultural, political.

Heavy metal diversifies and evolves fast in response to rapidly changing social and technological factors. These dynamics mirror the equally rapid changes in the ways modern human societies interact with the environment, and which are driven by the same factors (exemplified by the social-ecological systems dimension in Fig. 1). However, the interactions between people and the environment have reached dimensions that have become potentially detrimental to the planets capacity to support humankind (Rockström et al. 2009). Although it is highly uncertain how current patterns of human-environment interactions will unfold in the future, several social-ecological challenges, as those exemplified in this paper, will likely interfere with mankind’s welfare and the way humans interact with each other. Tribal societies have for thousands of years used music as an art form to express sentiments about and show emotional responses to phenomena observed in nature (Morely 2013). The counter-culture arts (Dada, Surrealism) of the 1920s in Europe both informed and were informed by socio-cultural-political revolutions, including rejection of traditional modes of authority and conservative societies. Those revolutions in the cultural landscape were connected partly to the music that helped drive those changes, as well as reflected those changes (LeBaron 2002). Similar dynamics can be observed in this Millennium, exemplified with the dynamics of heavy metal as a complex socio-musicological system.

Exploring apparently unrelated connections between different disciplines and art movements, such as surrealism and quantum physics (Parkinson 2008), or mental illness and political leadership (Ghaemi 2011), have shown opportunities for scientific inquiry that are not commonly practiced in academic circles. However, such lines of exploration open ways for a broader understanding of complex adaptive systems and have potential to inform our ability to understand, predict, and cope with their dynamics and change. It was the aim of this paper to show that the use of heavy metal as an art form can contribute to such an endeavor, exploring the use of the music to inform two different areas of scientific inquiry, complexity and sustainability science. While novel in itself, the independent discussions of the contribution of metal to the basic and applied sciences begs the question how metal as a socio-musicological system (Dimension 1; Fig. 1) and as a tool for environmental education (Dimension 2) are linked. Both dimensions entrain with each other and a plethora of other dimensions of social-ecological system dynamics not discussed in this paper to lead to the emergence of a broader social-cultural-environmental system (Fig. 1). The emanation of such a broader system cannot be explained simply by the sum of individual explorations of dimensions. In these dynamics the individual dimensions behave like actors that weave together and inform cultural, social and environmental facets in a broader complex adaptive system. Imagine a human body where the central nervous system modulates sensory, visual, auditory and olfactory stimuli. These processes are likely independent from each other and operate in distinct lobes of the brain, yet all are integral to the functioning of human live and coordinated by complex adaptive processes of the central nervous system. Music and its application to complexity and sustainability science can be similarly considered as independent; however, both are integral to human society and coordinated by broader social-cultural-environmental dynamics (Fig. 1). The patterns described for the brain and social-cultural-environmental systems are analogous and lay at the very heart of complex adaptive system dynamics described in this paper.

In conclusion, heavy metal, complexity theory, and sustainability science are not mutually exclusive. Together they become poietic in Plato´s sense; that is, bringing into existence the previously inexistent in endeavors to better understand the broader dynamics of and the multidimensional ramification of environmental change on complex systems of people and nature. Progress in human culture occurs in concert with, rather than independently of, art, science and the environment. Thus, there is need for integral models for envisioning and potentially dealing with the complexity of fast-changing social-ecological baselines that currently affect and are affected by humanity. Connecting the arts and sciences has enormous potential to provide learning opportunities about the known unknowns and the unknown unknowns associated with global change problems. Combining apparently unrelated entities, like heavy metal music and different scientific disciplines, may help people better envision and boost critical thinking about the complexity that drives the dynamics of broader social-cultural-environmental systems. Such approaches are necessary to facilitate citizens’ comprehension of the scientific and potential policy dimensions of environmental change, especially because it is the general public that bears the costs of a transforming planet.
